# Safety Implications of High-Field MRI: Actuation of Endogenous Magnetic Iron Oxides in the Human Body

**DOI:** 10.1371/journal.pone.0005431

**Published:** 2009-05-04

**Authors:** Jon Dobson, Richard Bowtell, Ana Garcia-Prieto, Quentin Pankhurst

**Affiliations:** 1 Institute for Science & Technology in Medicine, Keele University, Hartshill, Stoke-on-Trent, United Kingdom; 2 Sir Peter Mansfield Magnetic Resonance Centre, School of Physics and Astronomy, University of Nottingham, Nottingham, United Kingdom; 3 London Centre for Nanotechnology, University College London, London, United Kingdom; 4 Davy-Faraday Research Laboratory, The Royal Institution of Great Britain, London, United Kingdom; Clarkson University, United States of America

## Abstract

**Background:**

Magnetic Resonance Imaging scanners have become ubiquitous in hospitals and high-field systems (greater than 3 Tesla) are becoming increasingly common. In light of recent European Union moves to limit high-field exposure for those working with MRI scanners, we have evaluated the potential for detrimental cellular effects via nanomagnetic actuation of endogenous iron oxides in the body.

**Methodology:**

Theoretical models and experimental data on the composition and magnetic properties of endogenous iron oxides in human tissue were used to analyze the forces on iron oxide particles.

**Principal Finding and Conclusions:**

Results show that, even at 9.4 Tesla, forces on these particles are unlikely to disrupt normal cellular function via nanomagnetic actuation.

## Introduction

With the advent of advanced, high-field scanners for Magnetic Resonance Imaging (MRI), issues relating to the safety of these devices have recently been raised [1]–[3]. The European Union (EU) had recently proposed new safety standards for MRI which initially included limiting exposure to static magnetic fields to 2 Telsa (T). These have since been reconsidered. Most MRI units currently in use expose patients to fields of 1 to 1.5 T, however, newer clinical systems employ 3T magnets. In addition, 7 T whole-body scanners are becoming increasingly common for research use, exposing both patients and operators to high fields and, importantly, high field gradients.

The UK National Radiological Protection Board evaluated studies related to MRI safety and recommended sustained exposure limits for workers of 200 mT with 2 T being the ceiling for instantaneous, whole body exposure and exposure in the limbs is limited to 5 T [4]. These limits are based mainly on worker and patient reports of vertigo, nausea, metallic taste and phosphenes rather than on known biophysical mechanisms. However, the report also noted “the paucity of data on health effects of static magnetic fields”. The International Commission on Non-ionizing Radiation Protection are currently updating their guidelines on static magnetic field exposure, with a report due this year.

As field strength increases, it is necessary to evaluate the potential for interactions with human physiological processes. A recent study concluded that differences in the magnetic susceptibility between vestibular organs and surrounding fluid, as well as induced currents in vestibular hair cells, may be responsible for reports of vertigo-like sensations in subjects undergoing MR imaging in 7T research scanners [5]. Though this is an important study, the authors conclude that it is not likely that such effects would harmful.

In this study, we have taken a different approach and have evaluated the potential physiological effects of forces generated by high-field scanners on magnetic iron oxides, which are common in the brain and other organs [6], [7]. In 1992, biogenic magnetite (Fe_3_O_4_), a ferrimagnetic iron oxide, was discovered in the human brain [6] and studies since then have shown elevated levels of biogenic magnetite with both aging and Alzheimer's disease [8]–[11]. As the magnetic susceptibility of magnetite is more than two orders of magnitude higher than that of ferrihydrite (the form in which most iron in the body is stored within the ferritin protein) or goethite-like hemosiderin [12], its interactions with strong, static field gradients within the MR scanner merit investigation.

Both theoretical and experimental studies have shown that, under certain conditions, forces on biogenic magnetite particles have the potential to disrupt or alter the normal functioning of cellular ion channels [13]–[15]. This can lead to downstream effects on protein production and cell function [16], [17]. Therefore, we have investigated the forces acting on biogenic magnetite particles in the body under the field and gradient conditions present in a 7T whole body scanner and a 9.4T small-bore research scanner with the aim of determining the threshold for effects on cellular ion channels.

## Methods

**Figure 1:**
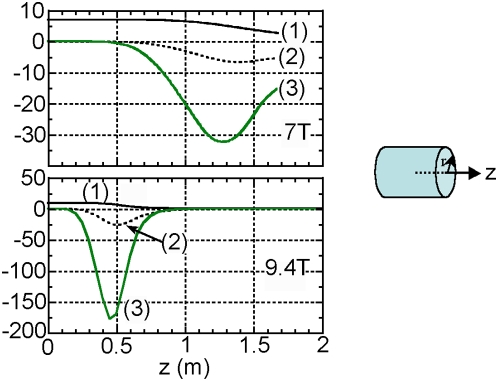
Magnetic field parameters for 7 T and 9.4 T MRI Scanners. Plots showing (1): 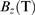; (2): 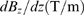; (3): 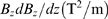 for the 7 T (top) and 9.4 T (bottom) MRI scanners.

Field profiles for a 7 T, 900 mm bore magnet and a 9.4 T magnet provided by the magnet manufacturers were used for the force calculations. Figure 1 shows the on-axis variation of B_z_, dB_z_/dz and the product of field and gradient B_z_×dB_z_/dz with z-co-ordinate for the two different magnets. The field profile data was then fed into a theoretical model to calculate the force on biogenic magnetite (Fe_3_O_4_) in the body based on both high-field and low-field susceptibility conditions as the subject is moved into the magnet [18], [19].

For conditions of low-field susceptibility (for magnetite, this is in fields below ∼200 mT), the following equation was used to model the forces acting on these magnetic iron oxides:
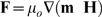
where **F** is the force on the particle, *μ_o_* is the magnetic permeability of free space, **m** is the induced magnetic moment on the particle and **H** is the magnetic field strength. For conditions of high-field susceptibility at which the magnetic particles would be saturated, forces were calculated according to:
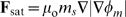
where *m_s_* is the intrinsic magnetic dipole moment, φ*_m_* is the magnetic scalar potential. In both cases the force scales with particle size, since the particle magnetisation **m** = V **M**, where V is the volume of the particle, and **M** is the volumetric magnetisation.

Forces were calculated for both the 7T whole body scanner and a 9.4T small-bore system for magnetite particle sizes of 100 nm and 500 nm diameter. Though most biogenic magnetite particles observed in magnetic extracts from brain tissue are smaller than this, particles in this size range have occasionally been observed [20]. Therefore, these particle sizes represent a “worst case scenario” for forces acting on naturally occurring magnetic iron compounds in the body.

It is also worth noting that, as can be seen from the above equations, in the absence of a field gradient, no force will be exerted on the particles. The gradient is therefore critical to the generation of the forces on these biogenic iron oxides.

## Results and Discussion

Most mechanosensitive ion channels operate close to the limits of thermal energy at body temperature (kT) and can be activated by applying direct forces of only a few picoNewtons [21], [22]. Applying such forces directly to the channel itself or via membrane deformation can disrupt the normal function of the channel, potentially forcing it open in response to the applied force [13]. Such a disruption can result in the over or under-expression of important proteins as well as osmotic stress due to changes in internal ionic concentrations. These effects can be potentially dangerous to the cell.

**Figure 2:**
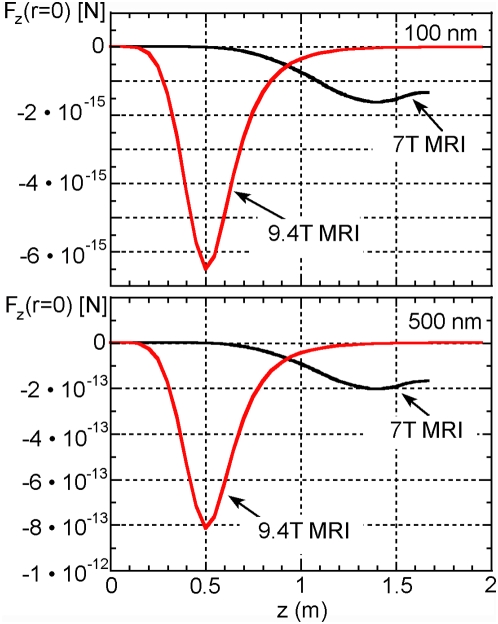
Force Plots. Plot of theoretical axial force on biogenic magnetite nanoparticles vs. axis position relative to the magnet centre for (a) 100 nm diameter magnetite particles and (b) 500 nm magnetite particles positioned on axis. Negative values indicate that the force is directed into the magnet.

Figure 2 shows the variation with axial position of the force experienced by 100 and 500 nm particles positioned on axis in the 7 and 9.4 T magnets. The negative sign indicates that the force is directed into the magnet as would be expected for paramagnetic material. The force peaks close to where the product of B_z_ and dB_z_/dz is largest.

Theoretical evaluation of the forces acting on 100 nm particles for both the 7T whole body scanner and the 9.4T research scanner reveals that any forces induced on the most magnetic iron compounds in the body would fall well short of the threshold for channel activation/disruption (Figure 2a). In the case of 500 nm biogenic magnetite particles, the 9.4T scanner produces forces very near this threshold (Figure 2b). However, in order to activate/disrupt ion channel functioning at this level of force, the particle would have to be directly coupled to the channel itself – even then it would fall just short of the activation threshold. Though this is a small-bore research scanner not meant for human imaging, 9.4 T scanners have been used to image the human head [23], [24].

As the availability of high-field MR scanners increases, it is important to evaluate the potential effects of these fields on various aspects of human physiology. A recent study by Atkinson et al. [24] examined cognitive performance and vital signs on patients exposed to a 9.4T field and determined these were not affected. The group concluded that these data suggest 9.4T imaging does not pose a health risk, although the parameters evaluated would only show an effect if it was an acute response to the field rather than a long-term response due to changes in cell function or general physiology.

After evaluating the fundamental physiological effects of high-field MRI on iron compounds in this study, it is apparent that even very high-field scanners will not likely initiate magnetic nanoparticle-mediated ion channel activation, even via relatively large magnetite particles. It is important to note that superparamagnetic iron oxides (SPIOs) are also used as contrast agents for MRI. As these particles are generally smaller than a few tens of nanometers, it is clear that forces acting on such particles will also fall well below the threshold of ion channel activation. Therefore, from a standpoint of iron oxide-mediated actuation of cellular bioeffects, MRI, even at very high fields and gradients, appears to present no discernable safety issues in our models. Experimental studies of longer duration and using varied *in vitro* and *ex vivo* models are needed to confirm the safety suggested by our theoretical results.
